# Less can be more: loss of MHC functional diversity can reflect adaptation to novel conditions during fish invasions

**DOI:** 10.1002/ece3.701

**Published:** 2013-08-22

**Authors:** Catalina Monzón-Argüello, Carlos Garcia de Leaniz, Gonzalo Gajardo, Sofia Consuegra

**Affiliations:** 1 Department of Biosciences, Swansea UniversitySwansea, SA2 8PP, United Kingdom; 2 IBERS, Aberystwyth UniversityPenglais Campus, Aberystwyth, SY23 3DA, United Kingdom; 3 Laboratorio de Genética, Acuicultura y Biodiversidad, Universidad de Los LagosOsorno, Chile

**Keywords:** Aquaculture escapes, biological invasions, *Oncorhynchus mykiss*, rainbow trout, rapid evolution, selection

## Abstract

The ability of invasive species to adapt to novel conditions depends on population size and environmental mismatch, but also on genetic variation. Away from their native range, invasive species confronted with novel selective pressures may display different levels of neutral versus functional genetic variation. However, the majority of invasion studies have only examined genetic variation at neutral markers, which may reveal little about how invaders adapt to novel environments. Salmonids are good model systems to examine adaptation to novel pressures because they have been translocated all over the world and represent major threats to freshwater biodiversity in the Southern Hemisphere, where they have become invasive. We examined patterns of genetic differentiation at seven putatively neutral (microsatellites) loci and one immune-related major histocompatibility complex (MHC class II-β) locus among introduced rainbow trout living in captivity (farmed) or under natural conditions (naturalized) in Chilean Patagonia. A significant positive association was found between differentiation at neutral and functional markers, highlighting the role of neutral evolutionary forces in shaping genetic variation at immune-related genes in salmonids. However, functional (MHC) genetic diversity (but not microsatellite diversity) decreased with time spent in the wild since introduction, suggesting that there was selection against alleles associated with captive rearing of donor populations that do not provide an advantage in the wild. Thus, although high genetic diversity may initially enhance fitness in translocated populations, it does not necessarily reflect invasion success, as adaptation to novel conditions may result in rapid loss of functional MHC diversity.

## Introduction

Human-mediated global change is causing the local extinction of some species but also the spread of others, resulting in novel species interactions and changes in ecosystem functioning. However, we are still far from understanding why some introduced species become invasive or whether successful invaders are also the most capable of transforming ecosystems (Wardle et al. 2011). Invasive species can trigger dramatic changes in native biodiversity, both at community and ecosystem levels and represent one of the main threats to global biodiversity (Gurevitch and Padilla 2004; Carroll 2007; Randi 2008). Understanding what makes some species flourish in novel habitats and become invasive has become a central question in conservation biology (Sakai et al. 2001). In most cases, invaders are able to persist at small initial population sizes before expanding and becoming invasive (Novak 2007). The initial invasion phase represents a critical time for the adaptation of species to local conditions (Sakai et al. 2001), and invaders may pass through demographic bottlenecks until suitable environmental conditions arise (Prentis et al. 2008). The ability of founders to adapt to novel conditions may depend on population size and environmental mismatch, but also on levels of genetic variation (Garcia-Ramos and Rodriguez 2002; Bell and Gonzalez 2009). Away from their native range, invasive species are likely to be confronted with novel selective pressures and may be expected to display different rates of neutral versus functional genetic variation to those of donor populations. However, the majority of invasion studies have only examined genetic variation at neutral markers (Uller and Leimu 2011), and the roles of neutral versus nonneutral (functional) genetic variation remain poorly known. Knowledge of genetic variation at neutral markers may not accurately predict adaptive potential or fitness (Reed and Frankham 2001, 2003) and may reveal little about how organisms adapt to novel habitats (Ashley et al. 2003). Neutral genetic variation is typically only weakly correlated with evolutionary potential (Leimu et al. 2006), and the scarcity of invasion studies employing functional markers makes it difficult to understand the relationship between genetic variation, establishment success, and fitness. For example, gene flow could facilitate establishment by bringing new variants that could counteract genetic diversity typically lost in small founder populations (Kolbe et al. 2008; Verhoeven et al. 2011), but it can also slow down adaptations to novel conditions, potentially reducing fitness of invaders (Kinnison et al. 2007). Clearly, better knowledge of how neutral and functional genetic variation affect establishment success is necessary for managing emerging threats to global biodiversity posed by an increasingly large number of invasive species.

Rainbow trout (*Oncorhynchus mykiss*) is one of the most widely translocated invasive fishes in the world, having been introduced to 90 different countries and being now present in all continents except Antarctica (Casal 2006). The species was originally introduced to Chile and other parts of the Southern Hemisphere for recreational fishing in the early 1900s (Basulto 2003; Garcia de Leaniz et al. 2010). This was followed by a second wave of invasions in the late 1980s with the exponential growth of the Chilean salmon industry (Gajardo and Laikre 2003). Nowadays, rainbow trout are established throughout most of Chilean Patagonia, where they tend to outgrow and outcompete native galaxiid fishes (Young et al. 2009, 2010).

In order to gain a better understanding of the role of genetic variation in determining establishment success during biological invasions, we examined variability at neutral loci (microsatellites) and at a functional, immune-related locus (major histocompatibility complex, MHC class II-β) among translocated rainbow in Chilean Patagonia. Along with sexual selection, pathogen-driven natural selection is involved in maintaining MHC diversity in vertebrates (Piertney and Oliver 2006; Dionne 2009; Spurgin and Richardson 2010), so combining a study of functional and neutral genetic markers should provide a better insight into the adaption of invasive species confronted with novel selection pressures. Our expectation was that, unlike in the northern hemisphere where salmonids are endemic and natural populations are often locally adapted (Garcia de Leaniz et al. 2007a,b; Fraser et al. 2011), exotic salmonids in the southern hemisphere should display a full adaptation continuum, depending on age and origin of populations. Thus, these should range from maladapted donor populations still held in captivity, to recent invaders only briefly exposed to natural conditions, to fully naturalized fish which have been living in the wild for several generations. Under the influence of natural selection, we would expect more functional (MHC) than neutral (microsatellites) differentiation among these groups. Such a scenario provides a rare opportunity to examine the patterns of rapid evolution during human-mediated range expansions (Uller and Leimu 2011) and to examine the roles of neutral and functional genetic variation in determining establishment success.

## Material and Methods

### Study populations

**Figure 1:**
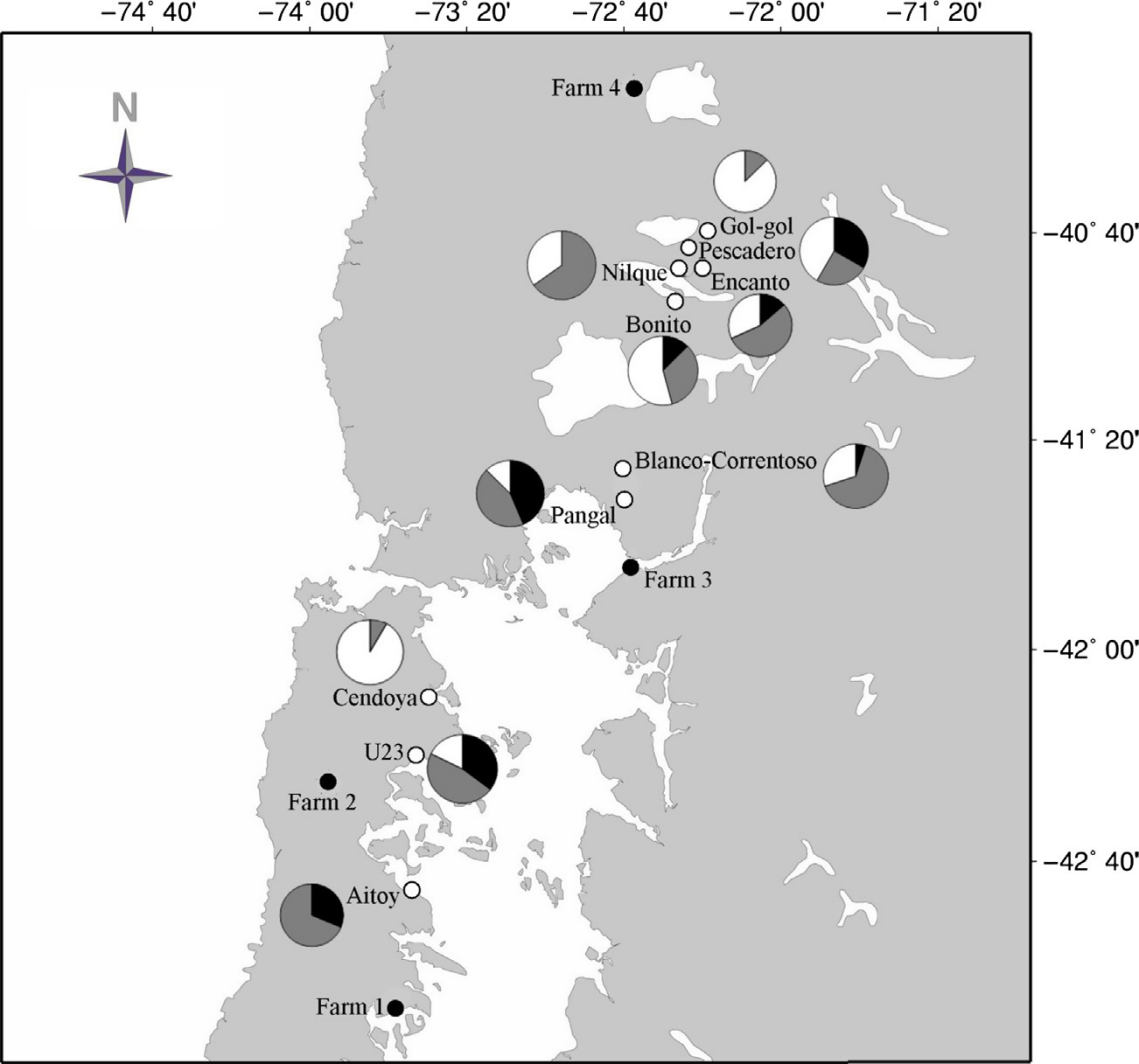
Sampling locations of rainbow trout (*Oncorhynchus mykiss*) populations in Chile. Free-living and farm populations are represented by open and closed circles, respectively. Each pie chart represents the proportion of escapees (black), hybrids (gray), and naturalized (white) trout across the 10 free-living populations. Map created using Maptool (http://www.seaturtle.org/maptool).

We analyzed 305 rainbow trout, including 211 wild (free-living) fish caught by single-pass electrofishing at 10 different rivers (stream order 1–3, and 100–150 m within high tide) and 94 farmed fish collected at four nearby fish farms in the Los Lagos Region of Chilean Patagonia during 2007–2009 (Fig. 1). These had been previously genotyped for seven microsatellites, and free-living individuals were genetically classified into three distinct groups according to admixture analysis: (a) naturalized (*n* = 85), (b) recent escapees (*n* = 33), and (c) hybrids between escapees and naturalized trout (*n* = 92) as described in Consuegra et al. (2011).

### Amplification, sequencing, and alignment of MHC class II fragments

A 237 base pair (bp) fragment of the exon 2 of the MHC class II-β gene, including part of the peptide-binding region (PBR), was amplified by polymerase chain reaction (PCR) using the forward primer B1RA (Miller et al. 1997) and a new reverse primer OnmyDAB_R4 (5'-TGTCCAGTATGGCGCTGTAG-3') designed from alignments of genomic DNA sequences of rainbow trout deposited in GenBank (GenBank Accession No. U34714.1–U34715.1). Approximately 80 ng of extracted DNA was used in 20 μL PCR mixes containing 0.2 μmol/L of each primer, 0.25 mmol/L of dNTPs, 0.5 U of *Taq* DNA polymerase (Bioline, London, U.K.), 1× buffer, and 2 mmol/L MgCl_2_. Thermal conditions consisted of 5 min initial denaturation cycle (94°C) followed by 35 cycles of 1 min at 94°C, 1 min at 57°C, 1 min at 72°C, and a final extension cycle of 10 min at 72°C. Amplified fragments were directly sequenced using the same PCR primers and resolved in a 3130 automated sequencer (Applied Biosystems, Foster City, CA). The resulting sequences were aligned using BioEdit 7.0.5.3 (Hall 1999) and compared with previously described rainbow trout sequences. Sequences that had not been previously described were cloned using the TOPO TA Cloning® Kit for Sequencing (Invitrogen, Carlsbad, CA) and six clones per individual were selected for forward and reverse sequencing.

### Genetic variation and population differentiation at functional (MHC) and microsatellite loci

Levels of neutral variation were estimated from the set of microsatellites previously amplified in Consuegra et al. (2011). For both microsatellites and MHC class II-β loci, allelic richness (*AR*) was calculated for the 14 study populations (10 free living and four captive) using the individual rarefaction method in PAST (Hammer et al. 2001) with a minimum sample size of 13 individuals. Observed and expected heterozygosities (*H*_o_ and *H*_e_), as well as departures from Hardy–Weinberg equilibrium, were assessed using Arlequin 3.5. We employed analysis of molecular variance (AMOVA; Arlequin 3.5) to explore how MHC variation compared between free-living and captive populations. The relationship between population diversity at MHC and neutral markers was investigated by examining the magnitude and significance of the Pearson correlation coefficient at *AR* and *H*_o_ and the extent of genetic admixture was calculated using Pielou's *J'* evenness index (Pielou 1966) as estimated in Consuegra et al. (2011).

We compared the genetic diversity (*AR* and heterozygosity) at microsatellite and MHC markers grouping the trout according to the time spent from introduction (captive fish, escapees, hybrids, and naturalized fish). *AR* (including 95% confidence intervals) was estimated in PAST using an adjusted sample size of 30 individuals/group. Differences in allelic diversity between pairs of groups were determined by resampling 1000 times with replacement a population with the same size as the smallest of the pair (Swanson et al. 2006). Differences in *H*_o_ among the four groups were tested using FSTAT 2.9.3.2 with 10,000 permutations, and group differentiation was assessed by pairwise *F*_ST_ and exact tests of allelic frequencies in Arlequin. We used analysis of covariance (ANCOVA) to compare patterns of change in genetic diversity (*AR* and *H*_o_) between both types of markers in relation to time spent as free living in the wild. To determine which MHC class II-β alleles were contributing most to group separation we used the SIMPER permutation approach (Clarke 1993) implemented in PRIMER 5.2.9 (Plymouth Marine Laboratory, U.K.) with contributing thresholds of 50%.

### Signatures of selection

Evidence of selection at MHC class II-β was examined via three codon-based approaches, comparing the relative abundance of synonymous to nonsynonymous sequence substitutions using MEGA v5.0 (Tamura et al. 2011), and using the fixed-effects likelihood (FEL) method with a significance level of 0.05 and the random-effects likelihood (REL) method with a minimum Bayes factor of 95 (DataMonkey; Wayne et al. 2010). In order to avoid the potential confounding effect of recombination, the presence of intragenic recombination was assessed using the single-breakpoint (SBP) analysis also implemented in DataMonkey.

## Results

### Genetic variation at neutral and functional markers

**Figure 2:**
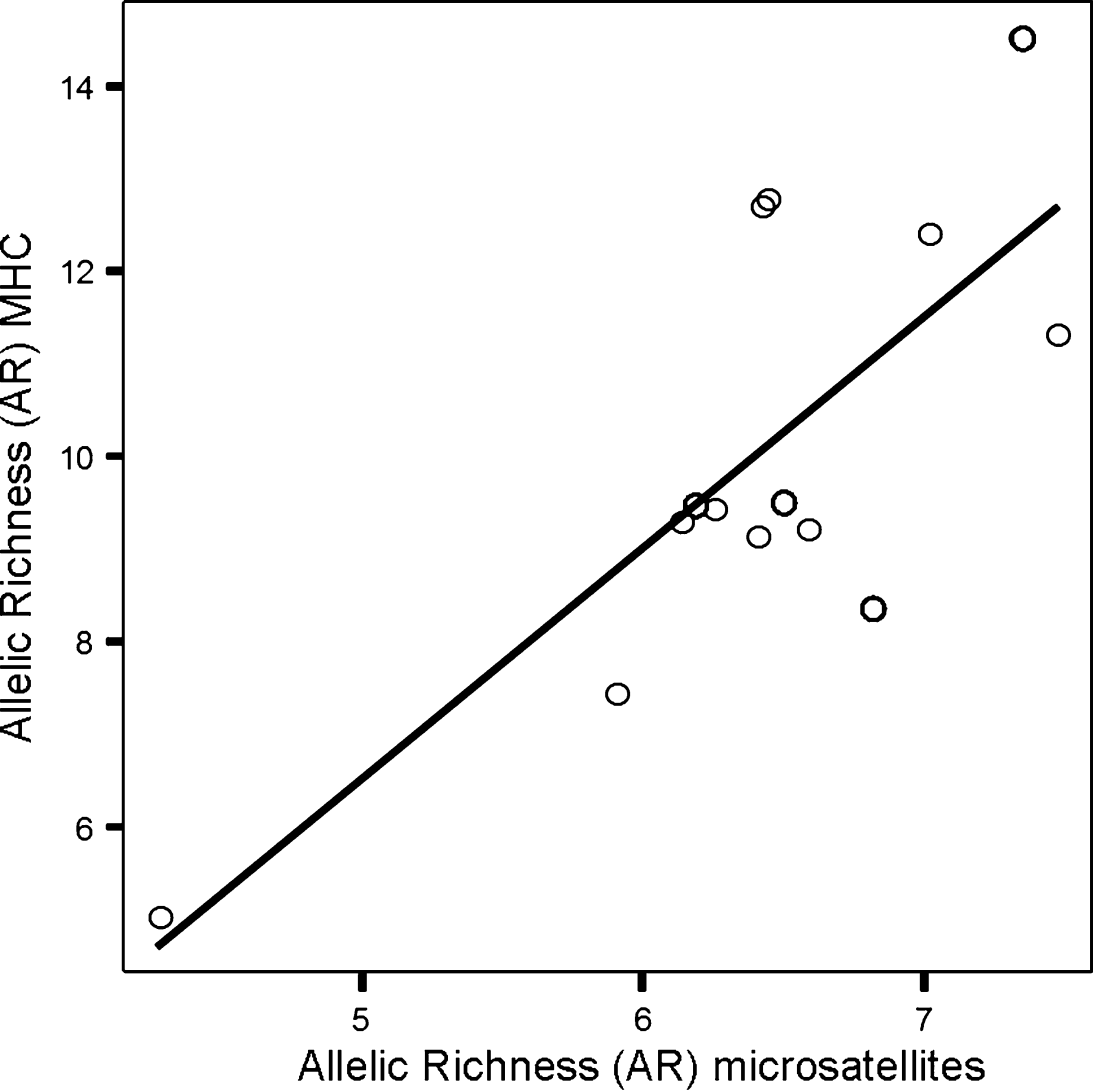
Relationship between MHC class II-β allelic richness (*AR*). Open and closed circles represent free-living and farm populations, respectively.

A total of 37 MHC class II-β alleles were identified, 12 of which represent novel sequences (GenBank Accession No. JN836529–JN836540; Fig. S1). Number of alleles per population ranged from *k* = 5 to *k* = 20 (Table 1). Genotype frequencies were in agreement with Hardy–Weinberg equilibrium in all populations after sequential Bonferroni correction (lowest *P* = 0.035). *AR* and observed heterozygosity (*H*_o_) were not statistically different between captive and free-living trout (*AR, P* = 0.702; *H*_*o*_ *P* = 0.343; Table 1). MHC *AR* was positively correlated with microsatellite *AR*, regardless of whether all individuals (*r* = 0.763, *P* = 0.002; Fig. 2A) or only free-living populations were considered (*r* = 0.795, *P* = 0.006). In addition, MHC *AR* was positively correlated with the extent of population admixture (*J'*) in all cases (all individuals *r* = 0.817, *P* < 0.001; free-living individuals *r* = 0.927, *P* < 0.001), suggesting that populations with higher neutral diversity and multiple genetic origins were also more diverse at the MHC class II locus.

**Table 1:** Diversity indices for the MHC class II-β locus and seven microsatellite (Microsat) markers in (A) 14 rainbow trout populations of Chilean Patagonia (including 10 free-living populations and four farms) and (B) individuals grouped according to the time spent in the wild (farmed, escapes, hybrids, and naturalized) based on admixture analysis

		*N*	*K*	*AR*	*H*_o_	*H*_e_	*J'*
(A) Free-living populations and farms							
Encanto	MHC IIβ	23	11	9.118	0.913	0.865	0.470
	Microsat		7.429 (2.225)	6.412 (1.567)	0.737 (0.139)	0.750 (0.092)	
Nilque	MHC IIβ	23	12	9.255	0.826	0.850	0.216
	Microsat		6.714 (1.380)	6.135 (1.030)	0.615 (0.171)	0.760 (0.046)	
Pescadero	MHC IIβ	24	16	12.752	0.958	0.928	0.817
	Microsat		7.429 (2.507)	6.455 (1.744)	0.643 (0.186)	0.753 (0.072)	
Bl-Corrent	MHC IIβ	20	11	9.422	0.842	0.824	0.668
	Microsat		6.875 (2.545)	6.253 (1.991)	0.627 (0.196)	0.744 (0.153)	
U23	MHC IIβ	17	14	12.410	0.941	0.914	0.977
	Microsat		7.286 (3.352)	7.023 (2.929)	0.739 (0.165)	0.798 (0.077)	
Aitoy	MHC IIβ	16	12	11.282	0.813	0.913	0.875
	Microsat		7.714 (2.870)	7.478 (2.618)	0.767 (0.179)	0.830 (0.069)	
Pangal	MHC IIβ	16	14	12.693	0.875	0.885	0.992
	Microsat		6.714 (2.430)	6.425 (2.179)	0.739 (0.137)	0.764 (0.119)	
Bonito	MHC IIβ	24	12	9.197	0.727	0.836	0.363
	Microsat		7.714 (2.628)	6.592 (1.901)	0.690 (0.089)	0.768 (0.065)	
Gol-Gol	MHC IIβ	24	9	7.409	0.792	0.822	0.216
	Microsat		7.143 (2.795)	5.906 (1.869)	0.678 (0.109)	0.716 (0.080)	
Cendoya	MHC IIβ	24	5	4.988	0.792	0.794	0.004
	Microsat		4.857 (1.464)	4.283 (1.100)	0.554 (0.266)	0.588 (0.191)	
Farm 1	MHC IIβ	24	13	9.458	0.958	0.839	0.004
	Microsat		7.286 (2.289)	6.188 (1.929)	0.696 (0.179)	0.725 (0.109)	
Farm 2	MHC IIβ	24	11	8.331	0.833	0.738	0.480
	Microsat		7.429 (2.507)	6.822 (2.024)	0.753 (0.216)	0.814 (0.071)	
Farm 3	MHC IIβ	22	20	14.506	0.909	0.928	0.555
	Microsat		8.429 (3.952)	7.348 (2.977)	0.760 (0.093)	0.790 (0.094)	
Farm 4	MHC IIβ	24	11	9.497	0.956	0.859	0.201
	Microsat		7.429 (2.637)	6.495 (2.117)	0.657 (0.248)	0.753 (0.090)	
(B) Individuals grouped by time spent in the wild							
Farmed	MHC IIβ	94	29	19.807 (0.452)	0.914	–	–
	Microsat		11.571 (6.133)	9.745 (4.063)	0.717 (0.159)	–	
Escapee	MHC IIβ	33	23	22.313 (0.188)	0.970	–	–
	Microsat		10 (4.761)	9.862 (4.605)	0.712 (0.111)	–	
Hybrid	MHC IIβ	92	25	17.473 (0.428)	0.810	–	–
	Microsat		11.571 (5.682)	9.351 (3.944)	0.673 (0.124)	–	–
Naturalized	MHC IIβ	85	20	13.630 (0.390)	0.833	–	
	Microsat		10.857 (5.014)	8.712 (3.115)	0.658 (0.118)	–	

*N*, sample size; *K*, number of observed alleles; *AR*, allelic richness; *H*_o_, observed heterozygosity; *H*_e_, expected heterozygosity; and *J'*, Pielou's *J'* evenness population admixture index; Bl-Corrent, Blanco-Correntoso. Standard deviation values are in brackets.

### Genetic differentiation at microsatellite and MHC loci

Genetic differentiation (*F*_ST_) based on MHC class II-β was 0.093 among all samples (*P* < 0.001). Differentiation was higher among trout living in captivity than among free-living populations (farm *F*_ST_ = 0.126; free-living *F*_ST_ = 0.070), with pairwise *F*_ST_ values ranging from 0.004 to 0.215 (Table S1). AMOVA analyses of free-living trout indicated that 2.8% of variation was explained by differences between locations, 5.7% was explained by differences among populations, and 91.6% of the variation was the result of differences within populations.

### Signatures of selection

**Figure 3:**
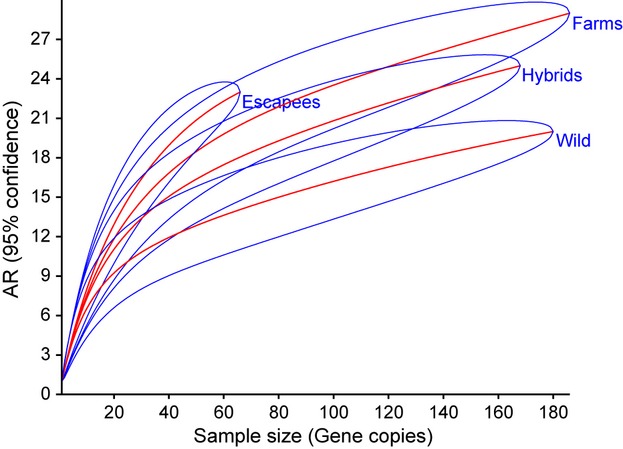
Allelic richness curves (*AR*; A) and 95% CI estimated using PAST for farmed, escapees, hybrids, and naturalized rainbow trout.

A total of 11 private MHC alleles were identified, eight in captive trout and one in each of the three free-living groups (Fig. S2). We found significant differences in MHC allelic diversity between hybrid and farmed trout (*P* = 0.030) and between naturalized and farmed trout (*P* = 0.001) but not between any of the other pairs (*P* > 0.9). MHC *AR* was lower in farmed fish than in escapees and then decreased with increasing time as free living (i.e., escapee *AR*>hybrid>naturalized; Table 1) and 95% confidence intervals did not overlap between naturalized fish and farmed or escaped trout (Fig. 3).

**Figure 4:**
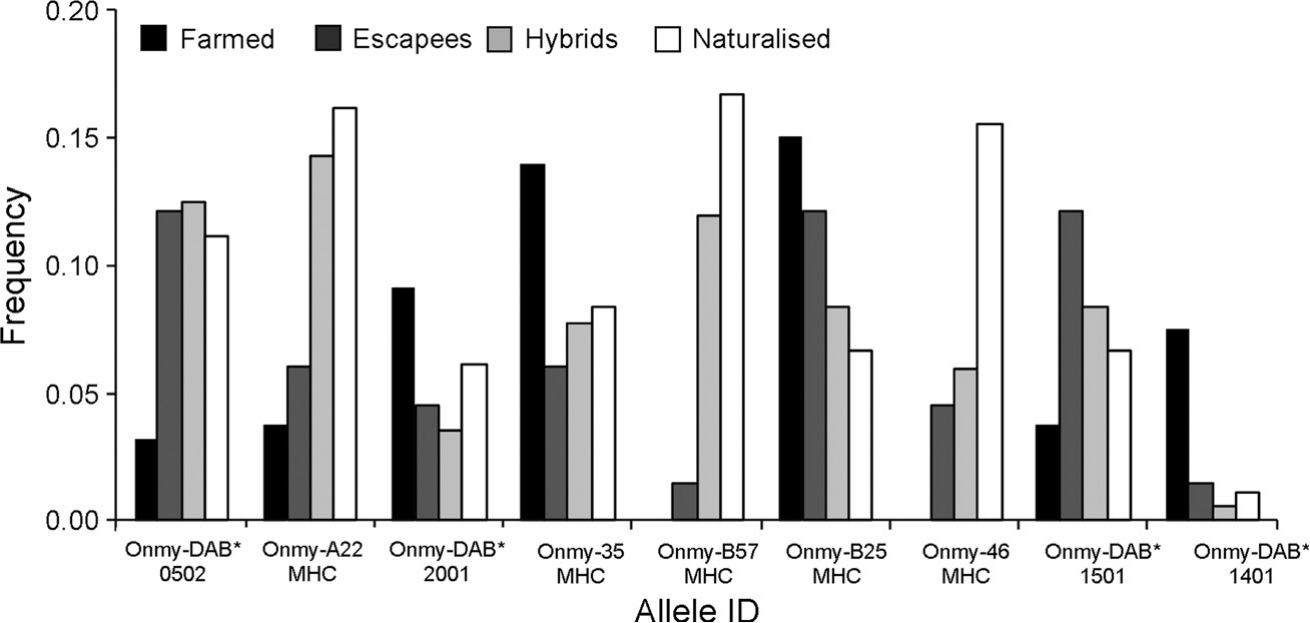
Frequency distribution of MHC class II-β alleles in farmed trout (black), recent escapees (dark gray), hybrids (light gray), and naturalized fish (white). Only the most discriminating alleles are shown (threshold value = 50%). All pairwise comparisons were significantly different except between hybrids and escapees and between hybrids and naturalized fish.

Significant differences in *H*_o_ at the MHC locus were also detected among the four groups (*P* = 0.033; Table 1) and, as with *AR*, *H*_o_ decreased with time spent in the wild although with a different pattern from *AR*, from 0.97 for escapees to 0.81 for hybrids and 0.83 for naturalized trout. A high proportion of *H*_o_ was lost in only one generation (from escapees to F_1_ hybrids). In contrast, no significant differences in *AR* (*F*_3,28_ = 0.273, *P* = 0.844) or heterozygosity (*F*_3,28_ = 0.861, *P* = 0.473) were detected among the four trout groups for neutral microsatellite loci, suggesting that diversity at these markers could be less affected by the time spent in the wild. ANCOVA analyses comparing the patterns of variation with time spent as free living between both markers revealed that the slopes between both markers were significantly different for *AR* (*F* = 168.5 *P* = 0.006) but not for *H*_o_ (*F* = 0.606, *P* = 0.518). All pairwise comparisons at microsatellite loci revealed significant genetic differentiation between the four groups of fish (*F*_ST_ and exact tests of allelic differentiation; Table 2). Differentiation at the MHC locus was significant between farmed and naturalized trout, but hybrids were not significantly different from escapees, or from fully naturalized trout (Table 2). In general, differentiation increased with time spent in the wild as free living for both neutral and functional markers. Results from SIMPER analysis indicate that most of the separation among the four groups of fish is mainly due to frequency differences at nine alleles (Table S2). Thus, alleles that were dominant among farmed trout (e.g., alleles 4 and 6) were less common among naturalized trout, whereas alleles that were frequent among naturalized trout (e.g., alleles 5 and 7) were absent among farmed fish and displayed intermediate frequencies among escapees and hybrid fish (Fig. 4).

**Table 2:** Pairwise *F*_ST_ values for seven microsatellite loci (above diagonal) and MHC class II-β (below diagonal) among trout grouped according to the time spent in the wild (farmed, escapee, hybrid, and naturalized)

	Farmed	Escapee	Hybrid	Naturalized
Farmed	–	**0.012**	**0.033**	**0.063**
Escapee	**0.012**^1^	–	**0.011**	**0.027**
Hybrid	**0.029**^1^	0.005	–	**0.013**
Naturalized	**0.048**^1^	**0.023**^1^	0.005	–

Significant values after sequential Bonferroni correction (*P* = 0.012) are in bold.

1 Significant pairwise comparisons based on the exact test of population differentiation.

The *Z*-test rejected the hypothesis of neutrality at the MHC class II-β locus (dN/dS = 4.2, one-tailed *P* = 0.002) and both the FEL and REL methods identified several sites to be under selection. FEL identified three positively selected and two negatively selected sites, whereas REL found 18 positively selected (three of them coincident with those identified by FEL) and two negatively selected sites. Of these, codon 47 was also identified as a potential recombination breakpoint.

## Discussion

Human-mediated species translocations are widely recognized as one of the main threats to global biodiversity (Gurevitch and Padilla 2004), but what makes invasions successful remains controversial. The role of genetic diversity in the successful establishment of introduced populations has long been discussed (e.g., Frankham 2005; Roman and Darling 2007), but the discussion has largely focused on variation at neutral markers, which does not always predict invasion success well (Uller and Leimu 2011). We suggest that one possible explanation of this inability to predict invasions is because novel selective pressures result in different rates of genetic variation at functional and neutral markers. We compared genetic variation at neutral microsatellite loci and an immune-related locus in invasive rainbow trout, a widely translocated species ranked by the IUCN among the world's 100 worst aquatic invaders. Although we found that neutral evolutionary forces (e.g., genetic drift and migration) play an important role in shaping immune-related MHC class II-β variation in rainbow trout during invasions, we also found evidence suggesting that natural selection could be acting on this locus. In this sense, several codons were identified to be under positive selection based on rates of nonsynonymous versus synonymous substitutions, three of them coincident between two different tests. Diversifying selection acting on the MHC could reflect the first stages of adaptation to local conditions, perhaps in response to pathogen-mediated selective regimes (Landry and Bernatchez 2001; Miller et al. 2010; Fraser et al. 2011), but could also be reflecting molecular signatures of selection generated in the original populations before the introduction, as they may take very long time to disappear even in the absence of selection (Garrigan and Hedrick 2003).

MHC allelic frequencies varied significantly depending on the time that fish had spent in the wild, and functional MHC (but not microsatellite) *AR* declined with increasing time spent in the wild as free living. Thus, rainbow trout held in captivity, as well as those that had recently escaped from aquaculture facilities (as evidenced from admixture analysis, Consuegra et al. 2011) were more similar in MHC allele frequencies to F_1_ farmed × free-living hybrids than they were to naturalized trout that had spent one or more generations in the wild. In contrast, all groups of trout differed significantly in microsatellite allelic frequencies, driven by neutral forces.

Population structuring analyses, particularly those based on *F*_ST_, can be problematic and must be interpreted with caution in invasive populations given that the assumption of equilibrium between mutation, drift, and gene flow may not be applicable (Fitzpatrick et al. 2012). However, in addition to MHC allele frequency variation, we also found changes in allelic composition with time spent as free living. Eight of the 11 MHC private alleles were detected among fish living in captivity, suggesting that alleles represented among free-living fish are only a subsample of those present in the farms. But we also found alleles present in the escapes and absent in the farms that are likely to have originated from farms not sampled in this study, suggesting that the number of alleles present among farmed fish but absent among naturalized trout could be even higher than our estimates. The strong differentiation observed between captive and free-living fish, as well as the similarity between hybrids and naturalized trout at the MHC class II-β locus might be indicative of selection. Selection may also explain the observed loss of MHC *AR* from escapees to naturalized trout, and perhaps account for the fact that most private alleles were found only among captive fish. Much of the heterozygosity was also lost from escapees to hybrids, suggesting that a large proportion of escapees may never reproduce in the wild, and that only a fraction of these may contribute genes to the hybrid generation. However, despite the small effective population size of naturalized rainbow trout populations in Chile (*N*_e_ < 100; Consuegra et al. 2011), they seemed capable of maintaining high levels of MHC class II-β variation (*AR* and heterozygosity), similar to those reported for natural populations at the species' native range (e.g., Aguilar and Garza 2006). This is in agreement with recent studies which also found high MHC variability among exotic salmonids in the region (Conejeros et al. 2011; Gómez et al. 2011). The positive association between MHC *AR* (but not heterozygosity) and population admixture suggests that populations founded from multiple origins not only display higher levels of neutral genetic diversity (Consuegra et al. 2011) but also higher variability at the MHC class II-β locus.

Thus, although sampling error and bottleneck effects may also account for the observed loss of diversity if only a small number of rainbow trout escaped from fish farms, this is unlikely to have been the case. Salmonid escapes in the region are substantial (Arismendi et al. 2009; Consuegra et al. 2011) and diversity at microsatellite markers did not vary with time spent in the wild, only MHC diversity did. Likewise, it could be argued that the observed differences in MHC composition between captive and naturalized fish may be due to temporal changes in broodstock, but our samples were collected from several populations over a relatively large area and during several years, and we found little evidence of temporal instability at microsatellite level.

Specific MHC alleles seem to confer resistance to a number of salmonid diseases (Langefors et al. 2001; Palti et al. 2001; Slierendrecht et al. 2001; Lohm et al. 2002; Grimholt et al. 2003), and disease resistance might explain better some of the MHC changes observed in our study. Salmonids in Chile are exposed to a suite of novel pathogens and diseases (Cabello 2007; Burridge et al. 2010; Torres et al. 2010) and some private alleles were clustered together, suggesting potential functional similarity (Fig. S2). Thus, the observed MHC differentiation between farmed and naturalized trout could have been the result of selection against some farm alleles that do not provide an advantage in the wild, possibly in relation to differences in mate choice and pathogen resistance between cultured and wild salmonids (e.g., Consuegra and Garcia de Leaniz 2008). The analysis of pathogen prevalence among the different trout groups with additional immune-related genes should help to test this hypothesis and further research appears warranted.

In summary, our results indicate that there is a significant decrease in MHC diversity and differentiation (but not in neutral variation) among invasive rainbow trout with increasing time spent in the wild. We suggest that despite the potential effects of bottlenecks and genetic drift associated with invasion and the massive number of escapees and continuous introgression of domesticated trout (Consuegra et al. 2011), pathogen-related selection and/or assortative mate choice might also play a role maintaining specific MHC alleles in naturalized populations. Thus, even if high genetic diversity can initially enhance fitness in translocated populations, it might not necessarily reflect invasion success if part of the functional genetic diversity was rapidly lost when invasive species adapt to novel conditions, a hypothesis that deserves further investigation.

## Supporting Information

**Figure S1.** Predicted protein sequence alignment of the 37 rainbow trout MHC class II-β sequences found in this study. A dot indicates amino acid identity with the consensus sequences. GenBank accession numbers are provided in brackets.

**Figure S2.** Neighbor-joining phylogenetic tree of predicted protein sequences of the 37 rainbow trout MHC class II-β alleles. Phylogenetic reconstruction and bootstrap values were obtained using a *P*-distance model in MEGA 5 (Tamura et al. 2011). Private alleles are denoted by: *farmed trout private alleles; **escaped trout private alleles; ***hybrid trout private alleles; and ****naturalized trout private alleles.

**Table S1.** Pairwise population differentiation (*F*_ST_) among Chilean rainbow trout populations for seven microsatellites (above diagonal) and the MHC class II-β locus (below diagonal). Significant values after sequential Bonferroni correction (*P* = 0.004) are in bold. B. Corren., Blanco-Correntoso.

**Table S2.** Alleles accounting for >50% of overall dissimilarity, ranked according to their contribution (C%: cumulative percentage), among the four groups (farmed, escapee, hybrid, and naturalized fish) based on SIMPER analysis (Clarke 1993).
