# Supplementary material for: Less can be more: loss of MHC functional diversity can reflect adaptation to novel conditions during fish invasions
Source: Ecol Evol. 2013 Aug 22;3(10):3359–68. doi: 10.1002/ece3.701 (PMC3797483; doi:10.1002/ece3.701)
Supplement: Supplementary file 1 [file ece30003-3359-SD1.doc]

**Supplementary material**

**Figure S1**. Predicted protein sequence alignment of the 37 rainbow trout MHC class II-β sequences found in this study. A dot indicates amino acid identity with the consensus sequences. GenBank accession numbers are provided in brackets.


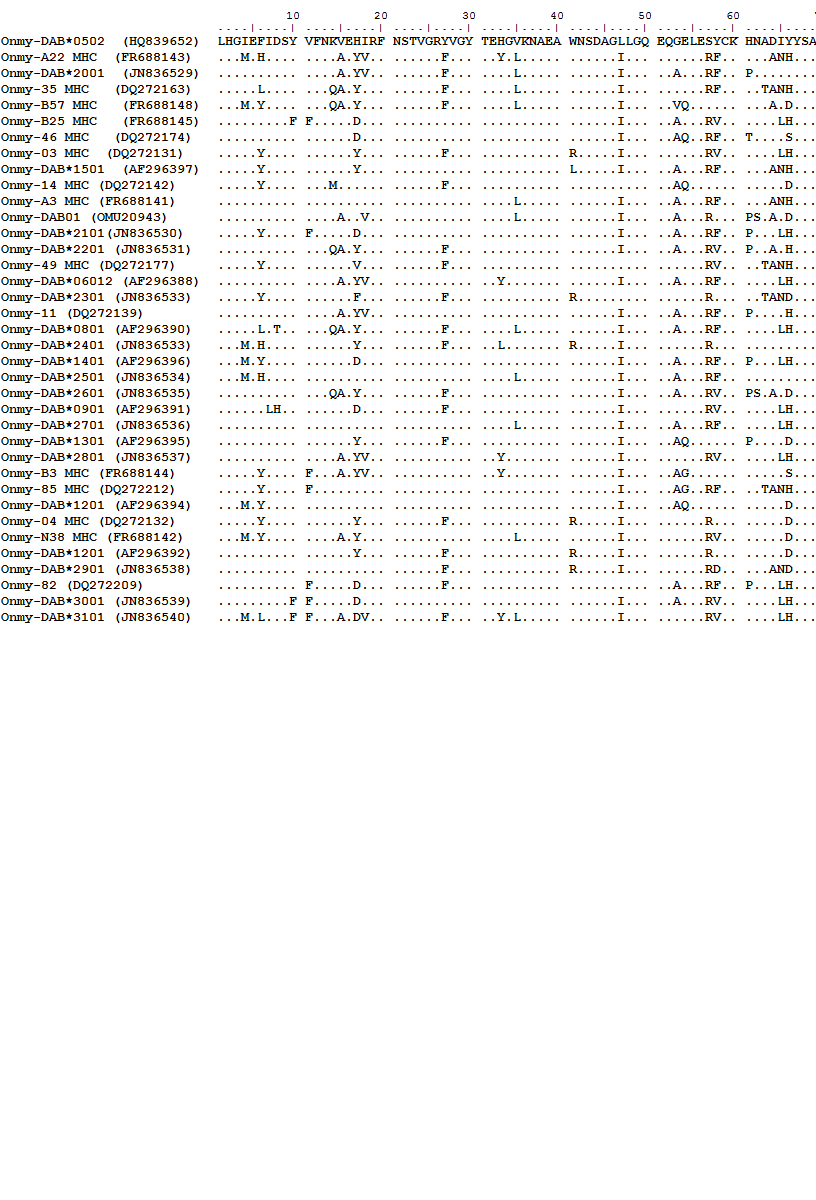


**Figura S2.** Neighbor-joining phylogenetic tree of predicted protein sequences of the 37 rainbow trout MHC class II-β alleles. Phylogenetic reconstruction and bootstrap values were obtained using a p-distance model in MEGA 5 (Tamura *et al.* 2011). Private alleles are denoted by : * farm trout private alleles; ** escaped trout private alleles *** hybrid trout private alleles and **** naturalised trout private alleles.


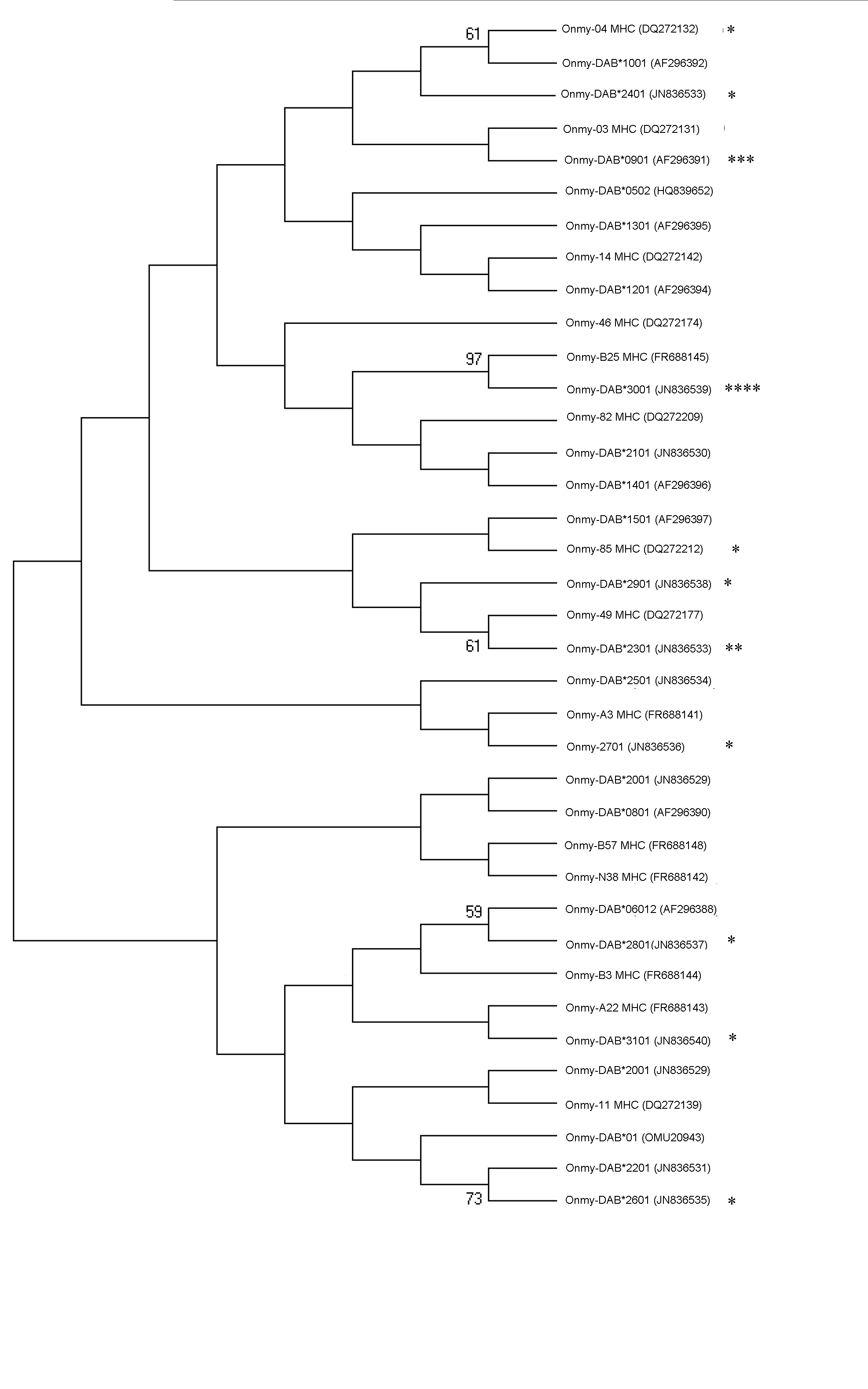


|  | Encanto | Nilque | Pescadero | B.Corren. | U23 | Aitoy | Pangal | Bonito | Gol-gol | Cendoya | Farm1 | Farm2 | Farm3 | Farm4 |
| --- | --- | --- | --- | --- | --- | --- | --- | --- | --- | --- | --- | --- | --- | --- |
| Encanto | **-** | **0.039** | **0.050** | **0.059** | **0.076** | **0.056** | **0.064** | 0.015 | **0.043** | **0.117** | **0.127** | **0.079** | **0.088** | **0.081** |
| Nilque | **0.055** | **-** | 0.027 | **0.043** | **0.062** | **0.047** | **0.070** | **0.043** | **0.069** | **0.122** | **0.115** | **0.074** | **0.073** | **0.060** |
| Pescadero | 0.026 | 0.023 | **-** | **0.059** | **0.063** | **0.054** | **0.070** | **0.061** | **0.064** | **0.134** | **0.094** | **0.080** | **0.071** | **0.069** |
| B. Corren. | 0.027 | **0.114** | **0.083** | **-** | **0.068** | **0.057** | **0.100** | **0.056** | **0.059** | **0.137** | **0.099** | **0.069** | **0.105** | **0.087** |
| U23 | **0.050** | **0.078** | 0.004 | **0.108** | **-** | 0.009 | **0.056** | **0.057** | **0.055** | **0.128** | **0.076** | **0.049** | **0.057** | **0.061** |
| Aitoy | **0.066** | **0.061** | 0.022 | **0.096** | 0.035 | **-** | **0.036** | **0.038** | **0.073** | **0.134** | **0.049** | **0.042** | **0.033** | **0.037** |
| Pangal | **0.068** | **0.109** | 0.023 | **0.099** | 0.018 | **0.062** | **-** | **0.047** | **0.090** | **0.150** | **0.081** | **0.063** | **0.040** | **0.048** |
| Bonito | 0.013 | **0.117** | **0.049** | 0.038 | **0.056** | **0.082** | **0.082** | **-** | **0.036** | **0.115** | **0.093** | **0.073** | **0.072** | **0.062** |
| Gol-gol | 0.020 | **0.073** | **0.064** | 0.032 | **0.099** | **0.071** | **0.109** | 0.039 | **-** | **0.079** | **0.127** | **0.102** | **0.110** | **0.108** |
| Cendoya | **0.123** | **0.071** | **0.059** | **0.168** | **0.079** | 0.050 | **0.113** | **0.154** | **0.118** | **-** | **0.205** | **0.184** | **0.185** | **0.182** |
| Farm1 | **0.120** | **0.140** | **0.055** | **0.160** | 0.035 | **0.099** | **0.039** | **0.125** | **0.162** | **0.140** | **-** | **0.089** | **0.074** | **0.068** |
| Farm2 | **0.176** | **0.098** | **0.091** | **0.215** | **0.108** | **0.119** | **0.135** | **0.203** | **0.203** | **0.073** | **0.153** | **-** | **0.038** | **0.065** |
| Farm3 | **0.089** | **0.091** | **0.049** | **0.085** | **0.041** | 0.034 | **0.068** | **0.099** | **0.115** | **0.114** | **0.091** | **0.136** | **-** | **0.054** |
| Farm4 | **0.094** | **0.123** | **0.064** | **0.115** | **0.067** | **0.088** | **0.089** | **0.095** | **0.129** | **0.107** | **0.094** | **0.187** | **0.079** | **-** |

**Table S1**. Pairwise population differentiation (F_ST_) among Chilean rainbow trout populations for 7 microsatellites (above diagonal) and the MHC class II-β locus (below diagonal). Significant values after sequential Bonferroni correction (p = 0.004) are in bold. Abbreviations: B. Corren., Blanco-Correntoso

**Table S2.** Alleles accounting for > 50% of overall dissimilarity, ranked according to their contribution (C%: cumulative percentage), among the four groups (farm, escapee, hybrid and naturalised fish) based on SIMPER analysis (Clarke 1993).

| **Farm vs Escapee** | |  | **Escapee vs Hybrid** | |  | **Farm vs Hybrid** | |
| --- | --- | --- | --- | --- | --- | --- | --- |
| Allele | C% |  | Allele | C% |  | Allele | C% |
| Onmy-B25 MHC | 11.17 |  | Onmy-DAB*0502 | 10.91 |  | Onmy-B25 MHC | 10.48 |
| Onmy-35 MHC | 20.64 |  | Onmy-A22 MHC | 20.56 |  | Onmy-35 MHC | 20.45 |
| Onmy-DAB*1501 | 28.61 |  | Onmy-DAB*1501 | 30.20 |  | Onmy-A22 MHC | 29.24 |
| Onmy-DAB*0502 | 36.30 |  | Onmy-B25 MHC | 39.76 |  | Onmy-DAB*0502 | 37.04 |
| Onmy-DAB*2001 | 42.99 |  | Onmy-B57 MHC | 46.83 |  | Onmy-B57 MHC | 43.49 |
| Onmy-A22 MHC | 47.94 |  | Onmy-35 MHC | 53.58 |  | Onmy-DAB*2001 | 49.74 |
| Onmy-DAB*1401 | 52.66 |  |  |  |  | Onmy-DAB*1501 | 55.68 |
| **Escapee vs Naturalised** | |  | **Farm vs Naturalised** | |  | **Hybrid vs Naturalised** | |
| Allele | C% |  | Allele | C% |  | Allele | C% |
| Onmy-A22 MHC | 10.37 |  | Onmy-35 MHC | 10.04 |  | Onmy-A22 MHC | 13.51 |
| Onmy-DAB*0502 | 20.73 |  | Onmy-B25 MHC | 19.81 |  | Onmy-B57 MHC | 26.37 |
| Onmy-46 MHC | 30.52 |  | Onmy-A22 MHC | 29.35 |  | Onmy-DAB*0502 | 37.63 |
| Onmy-B57 MHC | 40.03 |  | Onmy-B57 MHC | 38.27 |  | Onmy-46 MHC | 48.58 |
| Onmy-DAB*1501 | 49.17 |  | Onmy-46 MHC | 46.59 |  | Onmy-35 MHC | 56.57 |
| Onmy-B25 MHC | 57.98 |  | Onmy-DAB*2001 | 53.85 |  |  |  |

**References**

Clarke KR (1993) Non-parametric multivariate analyses of changes in community structure. *Australian Journal of Ecology* **18**, 117-143.

Tamura K, Peterson D, Peterson N, Stecher G, Nei M, Kumar S (2011) MEGA5: Molecular Evolutionary Genetics Analysis Using Maximum Likelihood, Evolutionary Distance, and Maximum Parsimony Method. *Molecular Biology and Evolution* **28**, 2731-2739.
